# Impaired Mitochondrial Dynamics and Bioenergetics in Diabetic Skeletal Muscle

**DOI:** 10.1371/journal.pone.0092810

**Published:** 2014-03-21

**Authors:** Ruohai Liu, Pengpeng Jin, LiqunYu, Ying Wang, Liping Han, Tong Shi, Xu Li

**Affiliations:** 1 Department of Anaesthesia, Second Affiliated Hospital, Wenzhou Medical University, Wenzhou, China; 2 Department of Physiology, Renji College, Wenzhou Medical University, Wenzhou, China; University of Iowa, United States of America

## Abstract

In most cells, mitochondria are highly dynamic organelles that constantly fuse, divide and move. These processes allow mitochondria to redistribute in a cell and exchange contents among the mitochondrial population, and subsequently repair damaged mitochondria. However, most studies on mitochondrial dynamics have been performed on cultured cell lines and neurons, and little is known about whether mitochondria are dynamic organelles *in vivo*, especially in the highly specialized and differentiated adult skeletal muscle cells. Using mitochondrial matrix-targeted photoactivatable green fluorescent protein (mtPAGFP) and electroporation methods combined with confocal microscopy, we found that mitochondria are dynamic in skeletal muscle *in vivo*, which enables mitochondria exchange contents within the whole mitochondrial population through nanotunneling-mediated mitochondrial fusion. Mitochondrial network promotes rapid transfer of mtPAGFP within the cell. More importantly, the dynamic behavior was impaired in high-fat diet (HFD)-induced obese mice, accompanying with disturbed mitochondrial respiratory function and decreased ATP content in skeletal muscle. We further found that proteins controlling mitochondrial fusion MFN1 and MFN2 but not Opa1 were decreased and proteins governing mitochondrial fission Fis1 and Drp1 were increased in skeletal muscle of HFD-induced mice when compared to normal diet-fed mice. Altogether, we conclude that mitochondria are dynamic organelles *in vivo* in skeletal muscle, and it is essential in maintaining mitochondrial respiration and bioenergetics.

## Introduction

Mitochondria are revealed to be highly dynamic organelles that show constant movement, fusion and fission [1], [2]. The overall morphology of mitochondria is maintained through the balance between mitochondrial fusion and fission. The role of mitochondrial dynamics is enabling content mixing of both mitochondrial matrix and membranous components among mitochondrial population [3], [4], which protects mitochondria from functional injury. Disruption of mitochondrial dynamics causes a series of diseases, including neurodegenerative disease [5], [6], cardiovascular disease [7], [8], and even cancer [9], [10].

In metabolically active skeletal muscle, mitochondria occupy ∼5% of the fibervolume and are rigidly located between bundles of myofilaments [11]. This specific arrangement makes the mitochondria show little motility which is required for mitochondrial fusion by colliding end-to-end or end-to-side [12] in many cells. Therefore, the question whether mitochondrial dynamics exists in skeletal muscle *in vivo* is raised. Until now, there are no direct experimental data to address this question and only several limited morphological results that indirectly showed that mitochondrial morphology was changed under pathophysiological conditions. Romanello V *et al.* showed that mitochondrial network is changed in atrophying muscle *in vivo* and inhibition of mitochondrial fission protects from muscle loss during fasting [13]. Further, mitochondria in fusion machinery deficient (MFN 1 and MFN 2 double knockout) skeletal muscle were fragmented into round spheres and accumulated into aggregates [4], indicating that the balance of mitochondrial fusion, fission and distribution are disrupted. Recently, smaller and shorter mitochondria were found in gastrocnemius skeletal muscle from both genetic-induced *ob/ob* and high-fat diet (HFD)-induced obese mice [14], indicating increased mitochondrial fission under these pathological conditions. Inhibition of mitochondrial fission by Drp 1 pharmacological inhibitor midivi-1 improved insulin signaling in C2C12 cells and obese mice [14]. Recent results showed that OPA1 appears essential for the normal adaptive response of skeletal muscle to training, manifested by blunted mitochondrial biogenesis in OPA1^+/−^ mice when they were adapted to exercise training [15]. Therefore, these data suggest that mitochondrial dynamics may be existed and plays an important role in maintaining the function of skeletal muscle.

To directly address whether mitochondrial dynamics exists in skeletal muscle *in vivo*, we expressed mitochondrial matrix-targeted photoactivatable green fluorescent protein (mtPAGFP) [16] in skeletal muscle using electroporation and examined mitochondrial dynamics in living anesthetic mice in real-time under confocal microscope. We found that mitochondria are quite dynamic and communicate with one another in skeletal muscle *in vivo* via mitochondrial fusion mediated by nanotunneling. Mitochondrial contents are transferred rapidly through mitochondrial network. Furthermore, the dynamic behavior is impeded in skeletal muscle in HFD-induced mice, accompanying with impairment of mitochondrial respiratory function and decrease of ATP content.

## Results

### Mitochondria are dynamic organelles in skeletal muscle *in vivo*

**Figure 1:**
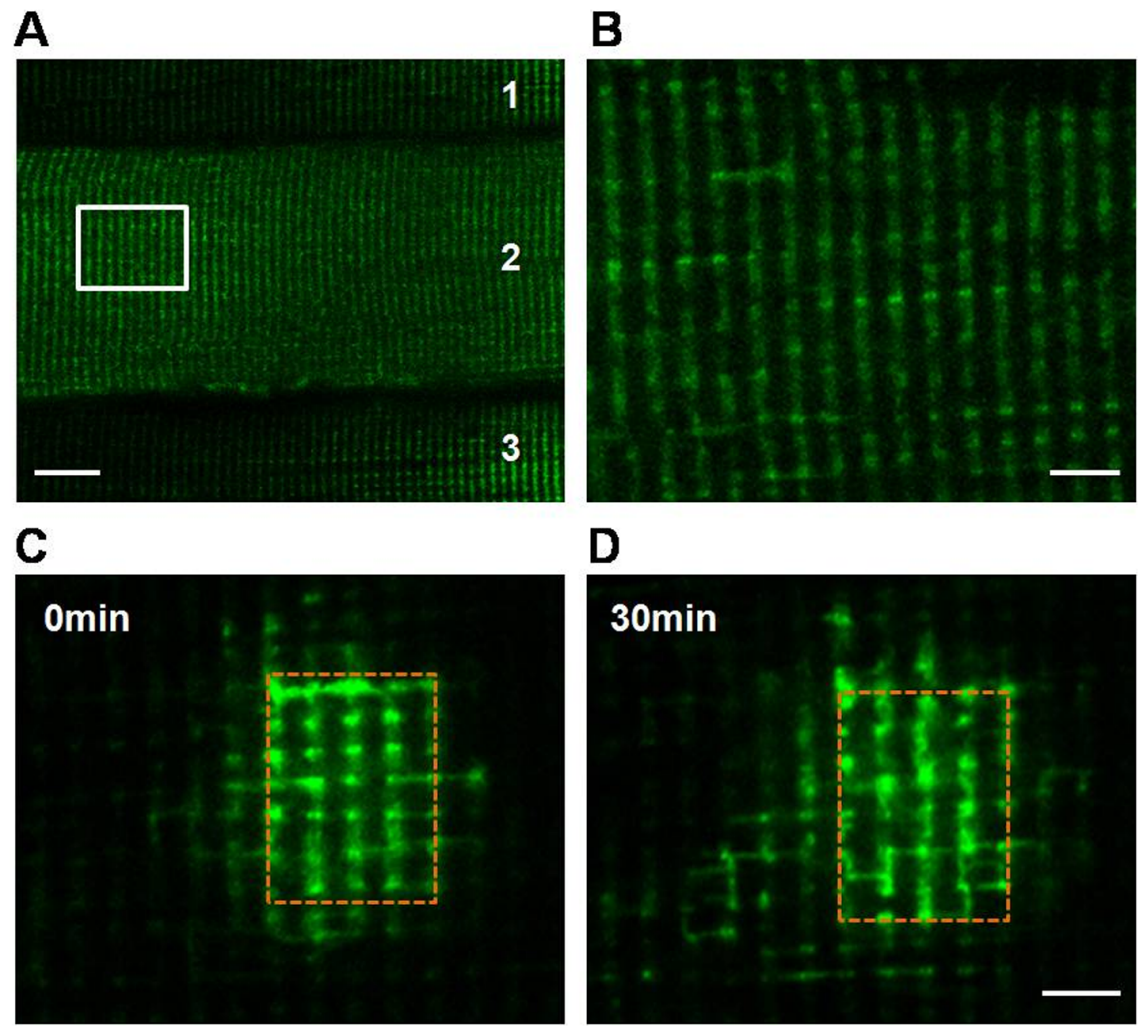
Dynamic mitochondria in skeletal muscle *in vivo*. A, Representative confocal image of mtPAGFP expression in skeletal muscle in anesthetic mouse. Numbers 1, 2, 3 represent three different skeletal muscle fibers. Scale bar: 20 μm. B, Magnification of the rectangle region in (A). Scale bar: 5 μm. C, Representative image showed the distribution and morphology of mitochondria in skeletal muscle after photoactivation of mtPAGFP. D, Redistribution of activated mtPAGFP 30 min after photoactivation. Dashed brown rectangle indicated photoactivated region. Scale bar: 5 μm.

Mitochondria in skeletal muscle are mainly organized in a highly regular “crystal-like” pattern [17] as a result of the restriction by myofilaments, yet until now, it is still unclear whether and how mitochondria communicate with each other in skeletal muscle especially *in vivo*. Here, mtPAGFP was applied to quantitative study of mitochondrial dynamics in skeletal muscle. We transfected mtPAGFP into white skeletal muscle fibers with electroporation, and examined mitochondrial dynamics in living animals under confocal microscope in real-time. As shown in Figure 1A, mtPAGFP was successfully expressed in muscle fibers 7 days after electroporation, but its expression was not uniform among different fibers (fiber 1, 2 and 3). Higher magnification of the image revealed that mitochondria in skeletal muscle were mainly arranged into ‘doublets’ along the transverse direction of the cell (Figure 1B), which is quite different from the arrangement of mitochondria in cardiomyocytes [18].

To investigate if mitochondria in skeletal muscle are dynamic organelles, a region of interest (ROI) was irradiated with 405-nm laser, and activated mtPAGFP showed about 30 times increase of fluorescence (Figure 1C). To our surprise, photoactivated mtPAGFP transferred to its neighboring non-activated regions 30 min later (Figure 1D and Movie S1), demonstrated by the evidently expanded area of fluorescence. Therefore, these data suggested that mitochondria are dynamic organelles in skeletal muscle *in vivo*, and mitochondrial contents can be transferred among mitochondria.

### Mitochondrial fusion in skeletal muscle through nanotunneling

**Figure 2:**
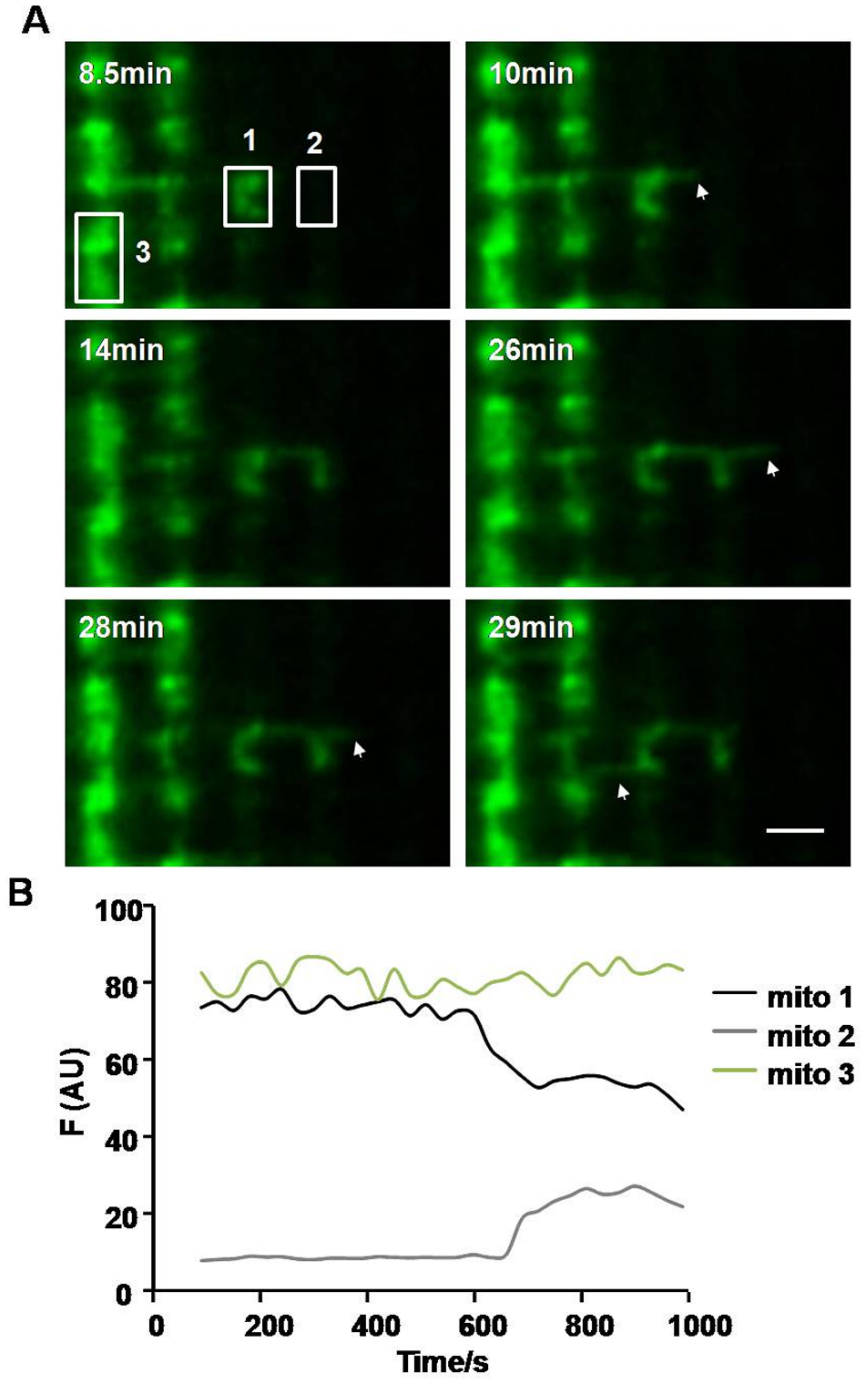
Mitochondrial fusion in skeletal muscle via nanotunneling. A, Confocal images showed the processes of extending of mitochondrial nanotubule and fusion with neighboring non-activated mitochondrion. Arrows: mitochondrial nanotubule. Scale bar: 2 μm. B, Time-course of fluorescence showed the fluorescence change during mitochondrial fusion.

Next, we explored how photoactivated mtPAGFP was transferred to its neighboring regions. We performed time-lapse experiments at an interval of 30 seconds. After lots of survey, we found that activated mtPAGFP could be propagated to its neighboring non-activated regions through mitochondrial fusion (Figure 2A). A thin mitochondrial filament extended from photoactivated mitochondrion 1 at the time point of 10 min, then contacted and fused with non-activated mitochondrion 2 at time point of 14 min (Figure 2A and Movie S2), manifested by the sudden decrease of fluorescence in mitochondrion 1 accompanying with fluorescence increase in mitochondrion 2 (Figure 2B), indicating that mitochondrial contents were exchanged between the two mitochondria. This kind of mitochondrial fusion was almost similar to nanotunneling in adult cardiomyocytes [19]. In addition, we found that fused mitochondrion 2 again protruded a thin mitochondrial nanotubule without fusion with another mitochondrion (28 min), and then the nanotubule retracted back to the main body (29 min) (Figure 2A and Movie S2). This kind of nanotunneling-mediated content exchange extensively exists in skeletal muscle *in vivo*, demonstrated by the fact that nanotunneling could be observed in nearly 80% recordings over a period of 30 min (a 6.0×6.0 μm^2^ area). The 3-dimentional reconstruction (Figure S1) of the nanotunneling further confirmed that the mitochondrial fusion was mediated by nanotunneling. Thus, these data showed that mitochondria in skeletal muscle are dynamic and can fuse and exchange contents with one another through nanotunneling.

### Interconnected mitochondrial network in skeletal muscle

**Figure 3:**
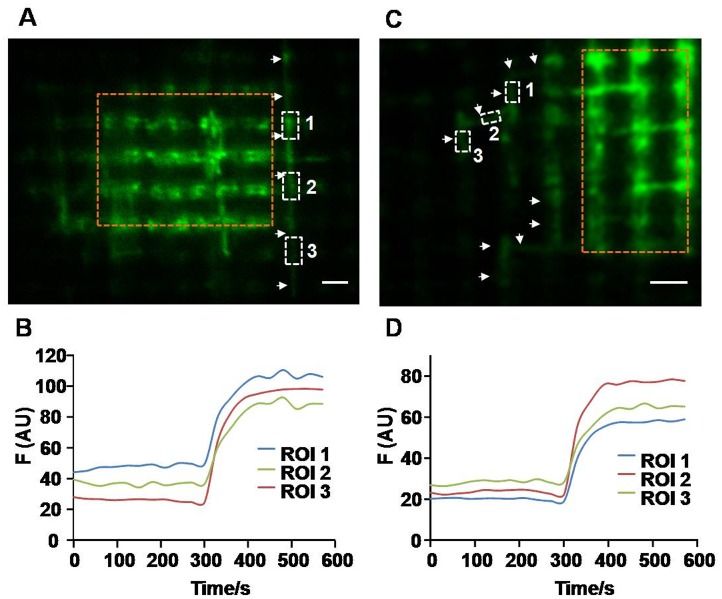
Mitochondrial network in skeletal muscle *in vivo*. A and C, Representative images showed the different morphologies of mitochondrial network soon after photoactivation of mtPAGFP in skeletal muscle. Arrows indicated the interconnected mitochondrial network. Dashed brown rectangle indicated photoactivated region. Scale bar: 2 μm. B and D, Time-course of fluorescence showed synchronous changes of fluorescence intensity within the mitochondrial network.

In addition to mitochondrial fusion, we also detected mitochondrial network in skeletal muscle, which allows for rapid propagation of mitochondrial contents among the mitochondrial population in the whole cell. Data in Figure 1C showed that the actual photoactivated area was larger than the region we drew, indicating that these mitochondria may be physically interconnected together. Here we dynamically recorded photoactivated region at the frequency of one frame every 30 seconds and found that activated mtPAGFP was quickly transferred to neighboring non-activated mitochondria nearly at the same time, which were almost up to more than 10 μm in length and spanned several mitochondrial bundles (Figure 3A). The traces of fluorescence clearly showed that the fluorescence of region 1, 2, 3 increased at the same time (Figure 3B), indicating that these regions are physically interconnected to form one mitochondrial network. In addition, other morphologies of mitochondrial networks have also been revealed in our experiments (Figure 3C and D).

### Characterization of HFD-induced obese mice

**Figure 4:**
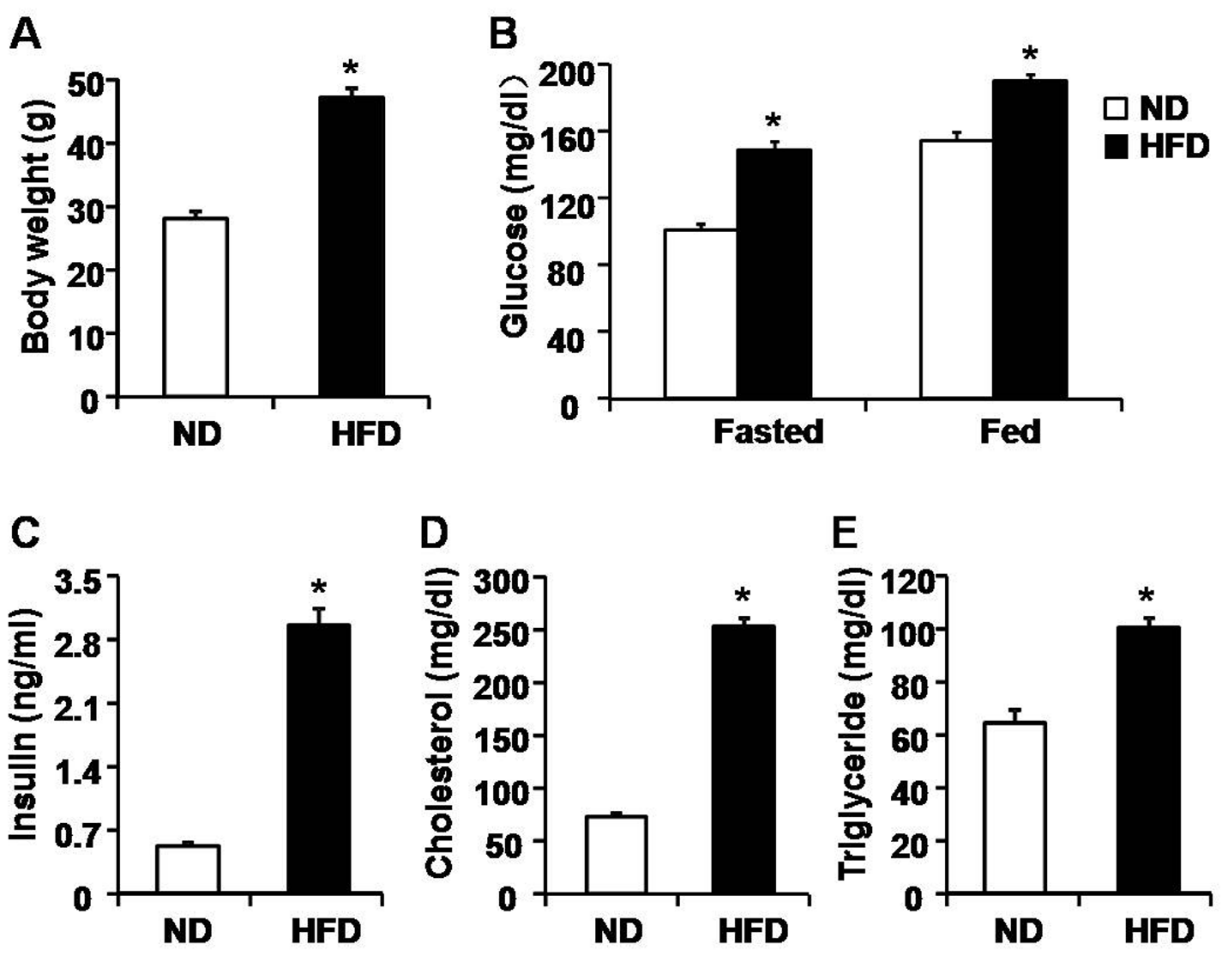
Biochemical characterizations of HFD-induced obese mice. A, Body weights of both normal diet (ND) and HFD-induced mice at the age of 44 weeks. B, Blood glucose contents both before and after food intake. C, D and E, Levels of serum insulin (C), cholesterol (D) and triglyceride (E) in control and HFD-induced mice. n = 10 mice for each group. *, P<0.05 compared to ND.

We next sought to explore whether there were some pathophysiological significances of mitochondrial dynamics in skeletal muscle. Here, we established HFD-induced mouse model to see whether mitochondrial dynamics changed under pathologic conditions. Body weight of HFD-induced mice (47.28±1.39 g) was significantly higher than that of normal diet (ND)-fed mice (28.15±1.12 g) (Figure 4A) after 40 weeks of treatment. The levels of blood glucose both before and after food-intake were higher in HFD mice than that in ND mice (Figure 4B). The levels of serum insulin and serum cholesterol were almost 3 times higher in HFD mice than that in ND mice (Figure 4C and D). In addition, triglyceride content in blood of HFD mice was also higher than that of ND mice (100.6±3.79 mg/dl in HFD *vs* 64.54±5.01 mg/dl in ND). Therefore, all these data indicated that the HFD-induced mice show obese phenotype.

### Impaired mitochondrial dynamics in HFD-induced obese mice

**Figure 5:**
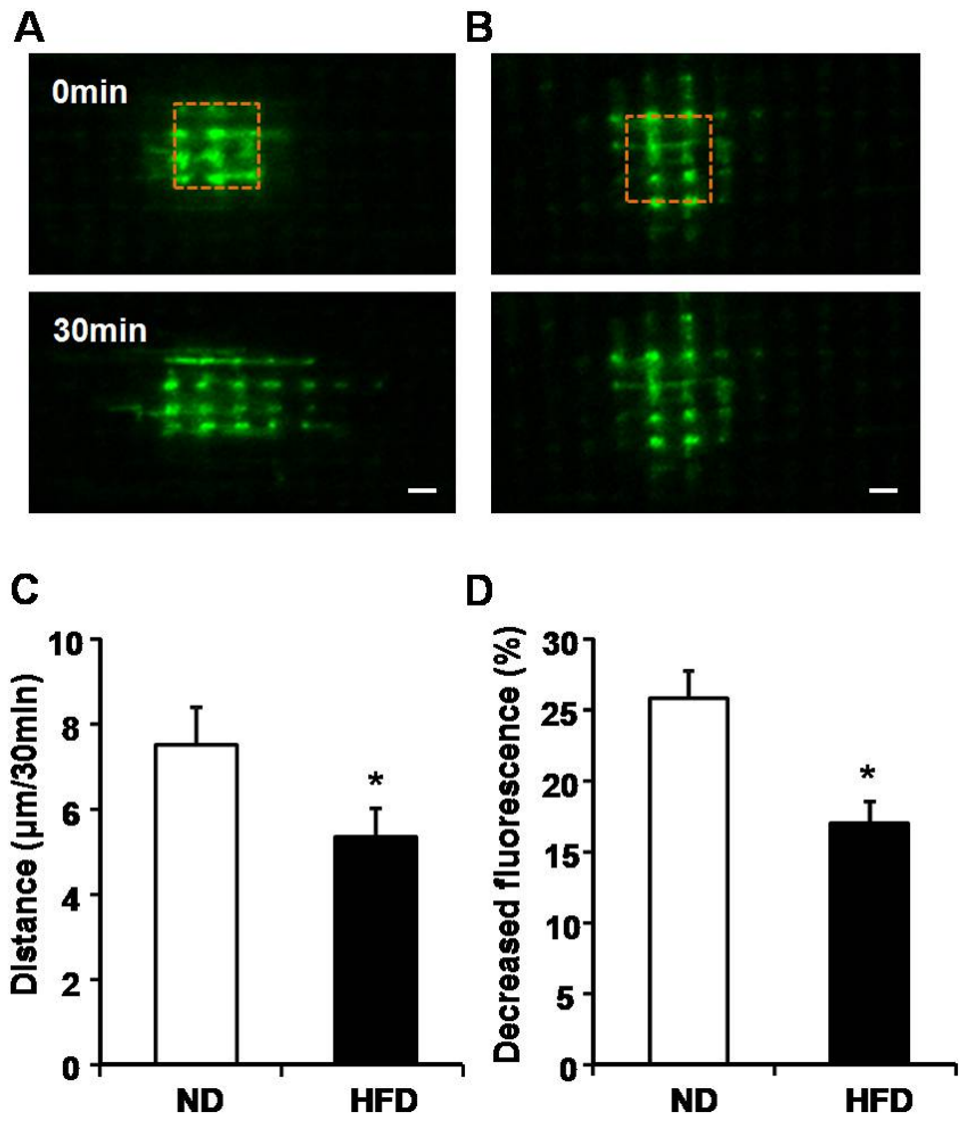
Inhibition of mitochondrial dynamics in skeletal muscle of HFD-induced mice. A and B, Representative confocal images showed the propagation of activated mtPAGFP in normal diet (ND) (A) and HFD-induced mice (B) 30 min after photoactivation. Dashed brown rectangle indicated photoactivated region with a size of 6.0 μm×6.0 μm. C and D, Statistic analysis on propagated distance (C) along longitudinal direction of the cell and decreased fluorescence of mtPAGFP of activated region (D) in (A) and (B). n = 6 mice for each group. *, P<0.05 compared to ND.

Next, we checked whether mitochondrial dynamics was changed in HFD-induced mice using photoactivation methods. We measured the propagation of activated mtPAGFP after photoactivation of a 6.0 μm×6.0 μm area (Figure 5A and B). As shown in Figure 5C and D, both the propagated distance along the longitudinal direction of the cell and decreased fluorescence of photoactivated region were declined in HFD-induced mice when compared to ND mice (distance: 7.51±0.88 μm in ND *vs* 5.36±0.67 μm in HFD; decreased fluorescence of photoactivated region: 25.79±1.92% in ND *vs* 17.03±1.52% in HFD), indicating that the content mixing was slowed down in HFD-challenged skeletal muscle. These data suggested that mitochondrial dynamics is compromised in HFD-induced obese mice.

### Disturbed mitochondrial respiratory function and decreased ATP production in HFD-induced mice

**Figure 6:**
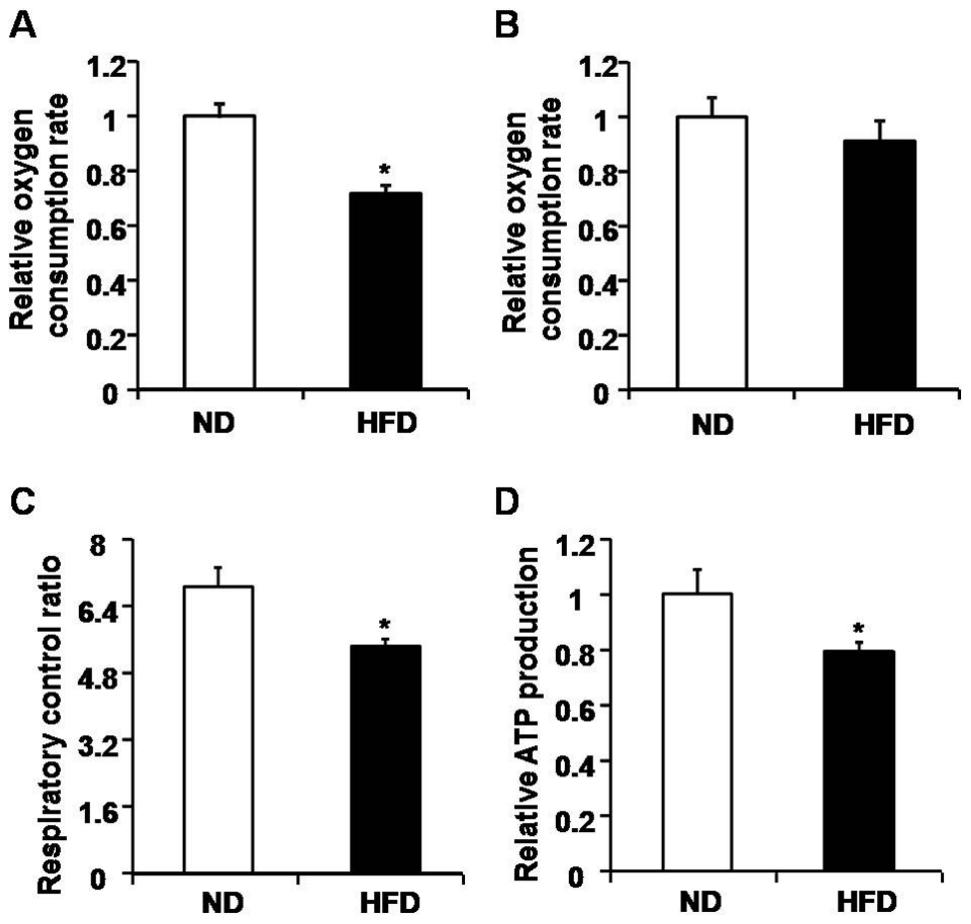
Impaired mitochondrial oxygen consumption and ATP production in skeletal muscle of HFD-induced obese mice. A and B, Relative oxygen consumption rate of state IV (B) and state III (A) in isolated mitochondria challenged by glutamate/malate and ADP. The rates in normal diet (ND) group was normalized to 1.00. n = 6 mice for each group. C, Respiratory control ratios (ratio of oxygen consumption rate of state III/state IV) in ND and HFD-induced mice. n = 10 mice for each group. D, Relative cellular ATP content in skeletal muscle from ND and HFD-induced mice. The ATP content in ND group was normalized to 1.00. n = 9 mice for each group. *, P<0.05 compared to ND.

Mitochondrial dynamics plays an important role in regulating mitochondrial function, including respiration and ATP homeostasis [4], [20]. Here we used oxygen electrode studies to measure the rate of oxygen consumption in isolated mitochondria from skeletal muscle. Substrate-stimulated maximal oxygen consumption rate stimulated by glutamate/malate and ADP (state III) was declined in HFD-induced mice (Figure 6A), but the rate of state IV did not change evidently (Figure 6B). Thus, respiratory control ratio was accordingly declined in HFD-induced mice (Figure 6C). These data together suggested that the coupling efficiency between electron transport and oxidative phosphorylation is hindered in HFD-induced mice. In line with the changes of mitochondrial respiratory function, we detected a significant decrease of ATP content in HFD-induced skeletal muscle compared with ND mice (Figure 6D). These data indicated that mitochondrial dynamics is impaired in skeletal muscle of HFD-induce mice, which may subsequently contribute to compromised mitochondrial respiratory function and bioenergetics, but whether the blunted propagation of mtPAGFP is as a result of disturbed mitochondrial fusion or mitochondrial networks needs further examination.

### Changes of machineries related to mitochondrial fusion and fission

**Figure 7:**
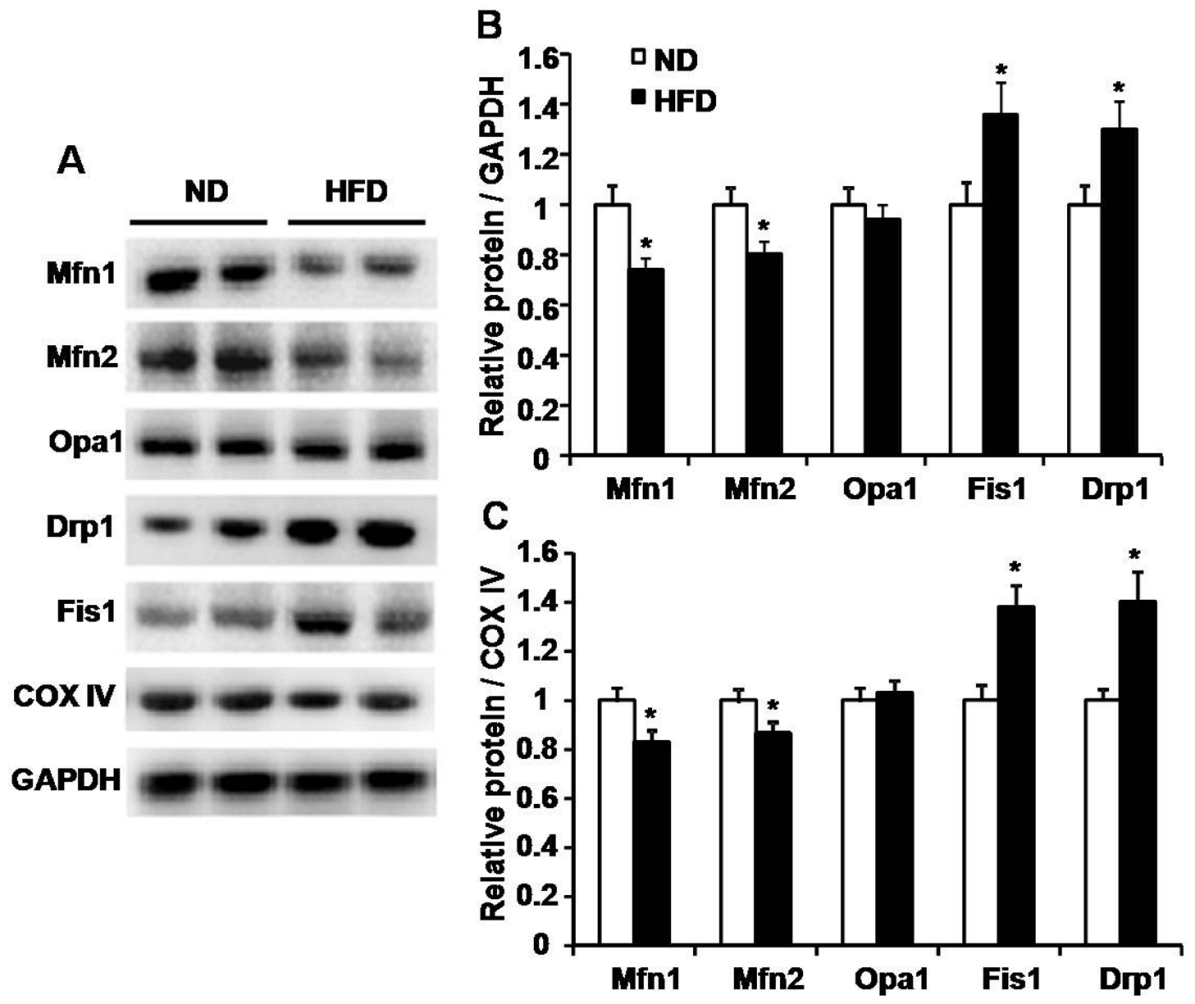
Expression of proteins regulating mitochondrial fusion and fission. A, Representative images of protein expression in skeletal muscle detected by western blot. B and C, The statistical results of normalized relative protein regulating mitochondrial fusion and fission/GADPH (B) or cytochrome c oxidase IV (COX IV) (C) ratio in skeletal muscle of HFD-induced mice when compared to control mice. n = 6 mice for each group. *, P<0.05 compared to ND.

Mitochondrial dynamics is tightly controlled by a series of “shaping proteins” in physiological conditions [1], [2]. We examined the expression of proteins regulating mitochondrial fusion and fission in both ND and HFD-induced mice. Compared to ND mice, proteins related to mitochondrial fusion MFN1 and MFN2 but not Opa1 were decreased, and the levels of Fis1 and Drp1 promoting mitochondrial fission were enhanced in skeletal muscle of HFD-induced mice (Figure 7A, B and C). These data suggested that HFD changes the expression profile of mitochondrial “shaping proteins” in skeletal muscle, which may contribute to the imbalance of mitochondrial dynamics and the impaired mitochondrial function.

## Discussion

Mitochondria are unique double membrane-bound organelles with their own genetic materials, mtDNA. Recent advances revealed that they play a central role in many cellular functions, including regulation of reactive oxygen species, calcium homeostasis and signal transduction, in addition to their most prominent roles in energy production. However, these functions are determined by their structures, morphologies and distributions, which are referred to mitochondrial dynamics. Dysfunction of mitochondrial dynamics is associated with a series of diseases ranging from developmental defects to cardiovascular and neurodegenerative diseases [5], [21], [22].

Our present study provides new insight into the mitochondrial dynamics in skeletal muscle *in vivo*. Ever studies have limited to investigate the dynamic behavior and molecular mechanisms on cultured cell lines, and little is known about the behavior of mitochondria *in vivo*. Here we found that mitochondria are dynamic organelles in skeletal muscle fibers *in vivo* for the first time with mtPAGFP, in spite of their compact and “crystal-like” arrangement between bundles of myofilaments [17]. These mitochondria dynamically communicated with each other through nanotunneling-mediated fusion, allowing them to exchange mitochondrial matrix contents manifested by the propagation of mtPAGFP. However, the propagation of mtPAGFP was blunted in HFD-induced mice, indicating that the dynamic behavior was inhibited in obese mice. This change was associated with the decrease of mitochondrial fusion proteins and increase of mitochondrial fission proteins. Finally, decreased mitochondrial dynamics was accompanied with impaired mitochondrial oxygen consumption and ATP production in HFD-induced mice.

Although mitochondrial dynamics has been described and extensively studied in C2C12 cells [23], [24], a cell model used for skeletal muscle study, these results cannot be directly applied to adult skeletal muscle, because both mitochondrial morphology and distribution are quite different between adult skeletal muscle cells and C2C12 cells. A main factor obstructing the advance of the study in mitochondrial dynamics in skeletal muscle is the lack of successful ways for detection of the dynamic behaviors, because it is hard to visualize the dynamics of mitochondria in skeletal muscle with conventional GFP imaging approach as a result of lack of mitochondrial mobility. Using photoactivatible technology, we observed intermitochondrial content transfer in living animals *in vivo* and directly demonstrated that mitochondria are dynamic organelles in skeletal muscle in real-time. With this approach, we also quantified the rate of mitochondrial communication as well as the detection of mitochondrial content exchange between fused mitochondria. In addition, *in vivo* imaging allowed us to visualize the real status and activities of mitochondria in living animals, which provides a unique and timely approach for the investigation of mitochondrial dynamics in both physiological and pathophysiological conditions.

Although we still did not detect mitochondrial movement in skeletal muscle, a prerequisite for conventional mitochondrial fusion, these mitochondria could communicated with each other by extending filamentous mitochondrial nanotubules—nanotunneling. With this manner, mitochondria can bypass the restriction of myofilament and “talk” with each other even if they are distant. These mitochondrial nanotubules are highly dynamic structures, demonstrated by the fact that the extending mitochondrial filament could fuse with neighboring mitochondria or retract to the main body. This kind of dynamic communication among mitochondria in skeletal muscle may protect the metabolically active cells from injury by preventing the accumulation of detrimental metabolites. However, whether these nanotubules in skeletal muscle are similar to that in cardiomyocytes is not known. The molecular mechanisms of nanotube formation and whether the process of nanotunneling-mediated fusion is the same as conventional mitochondrial fusion involved in both inner and outer mitochondrial membrane fusion merit further investigations. Furthermore, it should be noted that this technique has limitations in the detection of nanotubules. The diameter of nanotubules is reported to be 90 – 210 nm in adult cardiomyocytes [19], so the resolution of confocal microscopy (∼200 nm) may not be sensitive enough to catch all the nanotunneling events, if it is also in this case in skeletal muscle.

In addition, we also found that mitochondrial network exists in skeletal muscle, evidenced by the dynamic mtPAGFP study as well as static three-dimensional ultrastructure from EM picture [25]. Photoactivated regions were usually larger than the expected areas of illumination, demonstrating that these mitochondria were physically connected networks. These mitochondrial networks may be important for the contraction or functions of skeletal muscle. Because materials within one network could be transferred rapidly, it has been considered to be an effective way to transmit energy, oxygen and substrates from periphery to the core of muscle cells [26], [27]. Often, skeletal muscle fibers are large and range from 5 to 100 μm in diameter and from 1 or 2 mm up to even several centimeters in length [28], [29]. And these cells need huge energy supply when they contract, so gradients of oxygen and substrates exist from the periphery to the core of the cell, which may limit work performance of cell contraction. Thus, the rapid transport of oxygen, substrates and H^+^ from the edge to the central through mitochondrial network can well solve this problem.

More importantly, we found that mitochondrial dynamics is impaired in HFD-induced mice, which may provide us new insight to study the mechanism of obesity and diabetes. Skeletal muscle is the largest organ in the human body accounting for about 40% of the body weight, and recent studies have identified the central role of skeletal muscle in inducing whole-body insulin resistance and metabolic syndrome [30]. Now the detailed mechanisms and causal relationship between mitochondrial dysfunction and insulin resistance in obesity and diabetes are not clear.

Although the roles of mitochondrial shaping proteins in obesity and diabetes are somehow controversial, more and more evidences show that mitochondrial dynamics acts as a hub to bridge mitochondrial dysfunction and insulin resistance [31], [32]. The expression of MFN1, MFN2 and Drp1 was found not to be altered in obese subjects when compared with age-matched lean women by Holloway GP [33]. However, increased mitochondrial fission was reported to contribute to mitochondrial dysfunction and insulin resistance in skeletal muscle in obesity and type 2 diabetes [14]. A series findings by Zorzano's group also showed that altered expression of OPA1 and decreased expression of MFN2 participated in the development of obesity and type 2 diabetes in both patients and rodent models [32], [34], [35], [36], [37], which highlighted the importance of MFN2 in metabolism. Recently, a momentous advancement is that two groups simultaneously unmasked the involvement of MFNs in diet-induced obesity *via* the regulation of leptin resistance and systemic energy metabolism [38], [39]. In addition, expression of MFN2 improved HFD-induced insulin resistance and glucose homeostasis in liver [40], [41]. These findings are consistent with our results that HFD-induced mice showed a molecular shift from fusion towards more fission. Therefore, we next need to carefully examine the changes of mitochondrial dynamics during the development of insulin resistance, so that we could find better ways for when and how to intervene and treat obesity and diabetes through targeting mitochondrial dynamics.

## Materials and Methods

### Animals and dietary intervention

All animal experiments were performed in accordance with the National Institutes of Health Guidelines for the Use of Laboratory Animals, and were approved by the Wenzhou Medical University Committee on Animal Care. All surgery was performed under sodium pentobarbital anesthesia, and all efforts were made to minimize suffering. All mice were maintained under a 12-h light/dark cycle with free access to food and water. Male mice were grouped into normal diet and HFD groups and the HFD intervention started from 4 weeks. Mice were used for experiments after 40-week dietary administration.

### Electroporation and gene expression in skeletal muscle of adult mice

Electroporation of skeletal muscle *in vivo* was performed as previously described [42]. Briefly, anesthetized mice were injected with 10 μl of 2 mg/ml hyaluronidase dissolved in sterile saline in the hind limb. Then, 5–10 μg plasmid DNA (mtPAGFP) were injected into the same sites. Twenty min later, two electrodes were placed in parallel at the two sides of the injected leg. Twenty pulses of 100 V/cm and 20 ms were applied at 1 Hz using electroporation apparatus. The animals were used for experiments 7 days after transfection.

### Confocal microscopic imaging *in vivo*

The confocal imaging procedures are referenced to the method described by Fang H *et al* [43]. Mice were anesthetized with pentobarbital sodium (60 mg/kg body weight) by intraperitoneal injection. Skin near the transfection sites was shaved and sterilized with 70% alcohol. An incision was made and muscular mantle was carefully removed to expose gastrocnemius muscle (white muscle). All experiments have been performed using this kind of muscle fibers. Then gastrocnemius muscle was immersed in isotonic balancedsalt solution containing (in mM): 140 NaCl, 5 KCl, 2.5 CaCl_2_, 2 MgCl_2_ and 10 HEPES (pH 7.2) and placed on confocal microscope (Zeiss LSM 510) for imaging. Mice were placed on an isothermopad to maintain a constant body and muscle temperature. Transfected regions were first found using mercury lamp, and then confocal images were recorded with a 40×oil-immersion objective. After finding the transfected muscle cells, ROIs in cells were photoactivated with an intense 405-nm laser for designated durations. Then, time-lapse images were acquired by exciting at 488 nm and collecting the emission at >505 nm.

### Mitochondrial isolation and oxygen consumption measurement

Mitochondrial isolation from skeletal muscle were modified from the protocol described by Garcia-Cazarin ML [44]. Briefly, skeletal muscle was isolated quickly and then washed with ice-cold isolation buffer (in mM: 210 mannitol, 70 sucrose, 5 HEPES, 1 EGTA and 0.5 mg/ml BSA, pH 7.4), minced, and then digested with trypsin for 30 min. Digested muscle cells were homogenized on ice. The homogenate was centrifuged at 4°C for 10 min at 1000 g, and the supernatant was collected and further centrifuged at 4°C for 10 min at 10000 g. The pellet was re-suspended for functional assessment. The protein concentration of the mitochondrial preparation was determined by BCA Protein Assay Kit (Thermo scientific).

Mitochondrial respiratory function was measured with a Clark-type oxygen electrode. Mitochondrial protein (80–100 μg) was added into 1-ml respiration buffer (in mM: 225 mannitol, 75 sucrose, 10 KCl, 10 Tris-HCl, 5 KH_2_PO_4_, pH 7.2) at 25°C with 5 mM/2.5 mM glutamate/malate as substrate and 200 μM ADP for State III. Oxygen consumption rates of state III and state IV were normalized with mitochondrial protein content. Respiratory control ratio was referred to the ratio of state III to state IV respiratory rate.

### Measurement of ATP content in skeletal muscle

ATP production was determined with ATP Determination Kit (Invitrogen). ATP content was measured using freshly isolated skeletal muscle. Briefly, muscle tissues were excised and washed with ice-cold PBS, 80 mg tissue was homogenized in ATP extraction buffer (Tris/EDTA, 0.1 M/4 mM, pH 7.75). The homogenate was centrifuged at 12000 g for 5 min, the supernatant was collected, and ATP was measured according to the protocol provided by the kit. Protein concentration was determined using BCA Protein Assay Kit (Thermo scientific).

### Western blot analysis

The protein expression related to mitochondrial dynamics was measured by Western blot. The immunoblots were probed with anti-Opa1, anti-Drp1 (BD Biosciences), anti-Fis1 (Imgenex), anti-COX4, anti-MFN1 and Anti-MFN2 (Abcam) antibody overnight at 4°C followed by incubation with the corresponding secondary antibodies at room temperature for 1 h. Immunoblot results were visualized with ChemiDocXRS (Bio-Rad Laboratory) or LI-COR Odyssey® Infrared Imaging System. GAPDH was used as the internal loading control.

### Biochemical analysis of blood samples

Blood was collected from mouse tail vein and then concentrations of blood glucose, serum cholesterol and triglyceride were measured by an enzymatic assay according to the manufacturer's instructions (Applygen Technologies Inc., China). Insulin levels were determined as the protocol provided in an insulin ELISA Kit (Qcbio Science & Technologies, China).

### Statistics

Digital image processing and analysis were performed with Image J software (NIH). Statistical data are expressed as mean ± SEM and comparison between two groups was analyzed by Student's *t*-test. A p-value <0.05 was considered to be statistically significant.

## Supporting Information

Figure S1 **Contents transfer between mitochondria was observed in three-dimensional view.** A and C, Top view of mitochondria both before (A) and after content transfer (C) by three-dimensional reconstruction. B and D, Side view of mitochondria both before (B) and after content transfer (D) by three-dimensional reconstruction. Arrows indicate the mitochondrion communicating with photoactivated mitochondrion.
(JPG)

Movie S1 **Propagation of mitochondrial contents in skeletal muscle** ***in vivo*** **.**
(AVI)

Movie S2 **Nanotunneling mediated-mitochondrial fusion in skeletal muscle** ***in vivo*** **.**
(AVI)
