# Correction: Impaired mitochondrial dynamics and bioenergetics in diabetic skeletal muscle

**DOI:** 10.1371/journal.pone.0101265

**Published:** 2014-06-19

**Authors:** The PLOS ONE Staff

There is an error in the spelling of the third author’s name. The correct name is: Liqun Yu.

The correct citation is: Liu R, Jin P, Yu L, Wang Y, Han L, et al. (2014) Impaired Mitochondrial Dynamics and Bioenergetics in Diabetic Skeletal Muscle. PLoS ONE 9(3): e92810. doi:10.1371/journal.pone.0092810
