# Supplementary figures and images for: Impaired Mitochondrial Dynamics and Bioenergetics in Diabetic Skeletal Muscle
Source: PLoS One. 2014 Mar 21;9(3):e92810. doi: 10.1371/journal.pone.0092810 (PMC3962456; doi:10.1371/journal.pone.0092810)

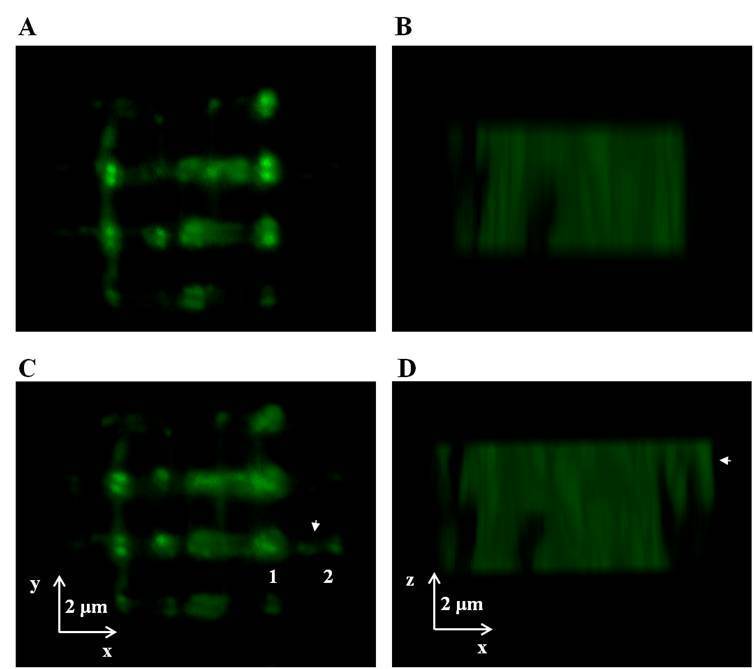

Supplement: Figure S1 — Contents transfer between mitochondria was observed in three-dimensional view. A and C, Top view of mitochondria both before (A) and after content transfer (C) by three-dimensional reconstruction. B and D, Side view of mitochondria both before (B) and after content transfer (D) by three-dimensional reconstruction. Arrows indicate the mitochondrion communicating with photoactivated mitochondrion. (JPG) [file pone.0092810.s001.jpg]
